# 744 Integrative Nursing framework in Burn ICU

**DOI:** 10.1093/jbcr/irac012.297

**Published:** 2022-03-23

**Authors:** Mini Thomas, Dina Friedman, Aemilio W Ha, Molly E Nunez

**Affiliations:** UCI Health, Orange, California; UCI Health, Orange, California; UCI Health, Orange, California; UCI Susan Samueli Integrative Health Institute, Orange, California

## Abstract

**Introduction:**

Patient and staff experiences are enhanced through a holistic and relationship-based approach of care. An integrative nursing (IN) framework was created utilizing various therapeutic modalities to augment healing for patients. Additionally, staff RNs were encouraged to utilize some IN modalities to aid in their own well-being. In 2019, the Burn ICU (BICU) was selected as one of five pilot units in the hospital to implement the IN framework. Post-implementation, staff were surveyed on their attitudes regarding the IN framework.

**Methods:**

The IN framework was created utilizing the IN principles and hospital policies. Two selected RN champions promoted this framework for staff and patients. In the BICU, over 95% of the RNs completed all five IN training modules: IN in Action, Acupressure, Clinical Massage, Aromatherapy, and Mind-Body skills. BICU was stocked with supplies, not limited to aromatherapy products, breathing ball, and massager chair. Additional resources were added including virtual reality (VR) and spiritual care. RNs documented interventions for pain/stress, anxiety/sleep, and nausea in the patient record. Post implementation, a self-report survey by RNs was utilized evaluated the IN framework.

**Results:**

A total of 27 surveys were collected from 29 available RNs. Aromatherapy, VR, therapeutic touch were the most utilized patient modalities, while staff mostly utilized the massage chair, aromatherapy, and deep tissue massager. Lack of time and personal discomfort were the highest reported barriers to utilization of the IN modalities.

**Conclusions:**

Creating and implementing an IN framework is achievable and can assist with improving patient and staff experiences in the hospital. Most staff and patients who utilized IN tools believed it provided benefits. Addition of IN modalities is the next step in providing patients with holistic care that treats not only the physical wounds but the whole patient.

